# Dr. Rogers, on the Use of Cold Water and Ice

**Published:** 1801-08

**Authors:** J. Rogers

**Affiliations:** Great Russel Street, Bloomsbury


### *To the Editors of the Medical and Phyſical Journal.*

Gentlemen,

If I can add any thing to what has been lately written and ſaid in favour of the external application of cold water in various diſeaſes of the human body, that may contribute in the leaſt to ſtrengthen the opinion of its utility, I ſhall conſider myſelf eminently gratified.

Having

Having had frequent opportunities of feeing the benefits arifing from fuch practice, I am authorifed to fpeak of it with a degree of confidence.

We are very much indebted to an ingenious phyfician for his publication on the fuccefs attending the external ufe of cold water in typhus, and I have heard its commendations often repeated in converfation, by feveral gentlemen who have experienced its efficacy in that diforder.

I cannot fay that I have ever feen it ufed in that fpecies of fever; but what I confider as equivalent, and what I have employed for many years with the ftrongeft marked evidence of every wifhed-for advantage in the fame diforder, is cold vinegar.

Typhus fever, about twenty-five years ago, was a very prevailing difeafe in St. Peterfburgh, but of late years, though often occurring, is much lefs frequent there; whether its appearing feldomer than formerly, is to be attributed to the improvement of the city, by being more built upon, or from drains dug fince that time to carry off the ftagnant water, or from the removal of part of the forefts in its neighbourhood, I will not pretend to decide; but certain it is, there is no comparifon between its frequency now and at the time above-mentioned.

In typhus fever, as I before obferved, I have been long in the habit of ufing cold vinegar, and that very liberally.

My method of employing it after previous evacuations, the fever being formed and characterized, was by dipping a napkin in it, and applying it to the abdomen, the patient being turned on his back for that purpofe; an operation feldom neceffary towards the end of the fever, the patient then, from weaknefs, generally lying in that pofture.

The application was frequently renewed, and at the fame time vinegar was fprinkled over the bed clothes, floor, and every part of the room, and a handkerchief or rag dipt in the fame, kept continually to the nofe, at which the patient generally expreffed figns of refrefhment. From this mode of practice, with free admiffion of air, clyfters of cold water and vinegar now and then injected, and plenty of cooling drink, with a convenient ufe of wine, I have experienced very great fuccefs, without ufing bark, a medicine to which, from its great reputation, I was formerly very much attached.

So great benefit has been derived from the exhibition of cold water in a variety of complaints to which the human conftitution is incident, that it cannot be too much infifted on and recommended. In typhus, from the experience of many of the firft practitioners in this kingdom, it has gained incontrovertible

 reputation, reputation, and in many other diseases it has been known to have been used with remarkable good effects.

It is in common use in northern climates in its extremest degree. In angina inflammatoria, by rubbing it on the outside of the throat in the state of ice. In acute rheumatism, by rubbing the part affected with it in the same aggregate form. In strangulated hernia, its use has been long and beneficially experienced; nor has it acquired less credit in ophthalmia, after proper evacuations.

I shall now relate a few cases where I have seen it succeed in an extraordinary manner.

A middle aged man, immersed in business to a degree of embarrassment, became insane; all the symptoms of mania in a short time came on; his pulse was full and strong, his eyes eager and inflamed, his ideas soaring to the summit of imaginary grandeur, fancying himself related to the greatest personages on earth, whilst his raving was incessant with continual insomnia. He was plentifully bled, purged, and vomited, used antiphlogistics of various denominations, and was kept some hours every day in the warm bath; but nothing abated the violence of his fury.

He became at length so refractory and unmanageable, that recourse was had to the strait waistcoat, medicines being given at the same time, without any good effect. I afterwards ordered his head to be shaved, and a large blister to be applied to it, which after remaining twenty-four hours was taken off, and four days afterwards, (the blistered place being then in a healing state,) his head was gently rubbed all over with a lump of ice; but the pain it seemed to produce would not allow me to continue its use long together; however, as soon as the uneasiness it produced appeared to subside, it was again had recourse to, and renewed every now and then till it manifestly incommoded him less. After some hours applying it, he begged we would release him from the strait waistcoat, promising to be quite obedient to our commands, and that he would use the ice himself; his request being complied with he kept his word, was quite composed, took the ice, and began rubbing his head with it, with more force than we had dared to do, and became so enamoured of the operation, that he was almost continually occupied in using it; and such was the result of his assiduity, that it soon procured tranquillity, induced sleep, and abated the violence of every symptom. He proceeded to get better, till he was enabled again to attend his business, which he was incautiously suffered to trouble himself too much about, and the consequence was a relapse, from which, I believe, he again escaped, under under the care of another gentleman; but on a third attack, I heard he died in England.

The second case I have to relate, is that of a Ruſſian nobleman labouring under a retention of urine, to whoſe aſſiſtance I was ſent for in the middle of night, in the depth of winter, when Reaumur's thermometer indicated 20 degrees of cold, correſponding with 11 of Fahrenheit's below zero. On the circumſtances of the caſe being related, I found that no urine had been diſcharged for thirty-ſix hours, although many means had been employed to promote it, ſuch as bleeding, purging, emollient and turpentine clyſters, with opium, &c. bougies and the catheter had likewiſe been attempted, but the reſiſtance at the neck of the bladder had been ſo obſtinate as to allow no admittance.

On my arrival, the patient complained of great pain about the region of the pubis; and the lower part of the abdomen was very much ſwollen and tenſe. His pulſe was yet full and hard, which encouraged me to repeat veneſection, although he had been pretty well evacuated before I ſaw him; but this affording him no relief, he became exceedingly anxious and impatient, imploring eaſe in the moſt plaintive manner. Now foiled, and almoſt at the ne plus ultra of my ſkill, I recollected the old trite adage of Celſus, "Anceps remedium, &c." and reſolved to adviſe his feet and legs to be put in cold water, a remedy I had ſomewhere heard mentioned or read of, as a powerful diuretic in obſtructed urine. He reluctantly complied with this propoſal; but on my promiſing him that he ſhould take them out in five minutes, he went through the experiment, ſitting on his bed's ſide.

The water he made uſe of was taken freſh from the river, the ice being broken for that purpoſe, and his legs were plunged into a pail full of it nearly up to his knees. He complained bitterly of the pain it occaſioned, but had reſolution enough to bear it till the expiration of the preſcribed five minutes, when he was releaſed from his purgatory, (his legs and feet wiped dry) and put to bed, ſoon after which, as he expreſſed himſelf, he felt a comfortable glow of warmth diffuſe itſelf over all his body, and in a few minutes afterwards called for a urinal, as he thought he could make water, which being given to him he by an endeavour forced out a few drops; this happy preſage enlivened my hopes, and emboldened me to give to him and the by-ſtanders ſtrong aſſurances of a favourable iſſue, which were verified by the event, for in about an hour, etſi guttatim, he filled the glaſs, and before morning had diſcharged a chamber-pot full. From this time the paſſage became free; and, as I was not his ordinary phyſician, I took my leave, and heard but but little of him afterwards; but from what I did hear, and having feen him frequently pafs the ftreets in apparent good health, I am inclined to think he received every benefit from the treatment he underwent that could be expected in fuch a cafe.

The third and laft cafe I fhall mention, was of a woman, extremely bilious and low fpirited, who, though her condition in life was above mediocrity, unfortunately indulged herfelf too much in the ufe of fpirituous liquors; the confequence of which was frequent hæmorrhagy from the nofe, in general not difficult to ftop by the common methods ufed for that purpofe, as vinegar, ice, &c. to the temples, noftrils and forehead, but on greater diffolution of the blood, the bleeding was more difficult to arreft, and on one occafion became fo alarming as to make me apprehenfive for the patient's life.

All the means adminiftered on former attacks were now practifed in vain, the bleeding was not to be ftopped by any of them, but continued to flow in an abundant ftream for feveral hours, till the ftrength of the body was reduced to a formidable ftate of weaknefs. No time was now to be loft, the hæmorrhagy muft be fuppreffed, or the patient fink from inanition. The only probable mean of fuccefs that I could think of was ice, which, by its fedative power, might leffen the impetus of the blood, by diminifhing the action of the heart and arteries; and here I was not difappointed, for on ordering ice to be applied to the cheft, abdomen, and vagina, in a few minutes the difcharge of blood from the nofe was checked, which, from flowing in a ftream, began now to efcape only by drops, and in a few hours the hæmorrhagy entirely ceafed, the ice being occafionally repeated till that ceffation had taken place. No bleeding at the nofe happened again in this patient; but from weaknefs brought on by the great evacuations fhe had experienced, and from perhaps a difeafed liver, fhe died dropfical about fix months after the period fpecified.

I could add many more inftances of the benefits accruing from cold applications, but am afraid I have trefpaffed too much on your conveniency by what I have already faid.

*Great Russel Street, Bloomsbury.* I am, &c.

